# *Plasmodiophora brassicae* Inoculum Density and Spatial Patterns at the Field Level and Relation to Soil Characteristics

**DOI:** 10.3390/pathogens10050499

**Published:** 2021-04-21

**Authors:** Andrea Botero-Ramirez, Sheau-Fang Hwang, Stephen E. Strelkov

**Affiliations:** Department of Agricultural, Food and Nutritional Science, University of Alberta, Edmonton, AB T6G 2P5, Canada; abotero@ualberta.ca (A.B.-R.); sh20@ualberta.ca (S.-F.H.)

**Keywords:** clubroot, geostatistics, epidemiology, spatial patterns, patchiness

## Abstract

Clubroot, caused by *Plasmodiophora brassicae*, is an important soilborne disease of the Brassicaceae. Knowledge of the spatial dynamics of *P. brassicae* at the field level and the influence of soil properties on pathogen spatial patterns can improve understanding of clubroot epidemiology and management. To study the spatial patterns of *P. brassicae* inoculum density and their relationship to different soil properties, four clubroot-infested fields in central Alberta, Canada, were sampled in 2017 and 2019, and *P. brassicae* inoculum density, soil pH, and boron, calcium, and magnesium concentrations were quantified. Spatial autocorrelation of the inoculum density was estimated for each of the fields in both years with the Moran’s *I* and semi-variograms. A Bayesian hierarchical spatial approach was used to model the relationship between *P. brassicae* inoculum density and the soil parameters. Patchiness of the pathogen was detected, with most patches located at the field edges and adjacent to the entrance. Infested patches grew in size from 2017 to 2019, with an average increase in diameter of 221.3 m and with this growth determined by the maximum inoculum density and active dispersal methods such as movement by machinery and wind. Soil pH, boron, calcium, and magnesium concentrations were not found to have an important effect on the inoculum density of *P. brassicae.*

## 1. Introduction

Canola (*Brassica napus* L.) is one of the most important field crops in Canada, contributing C$26.5 billion annually to the national economy [1]. In 2019, the crop was grown on approximately 8.6 million hectares, mostly in the western provinces of Alberta, Saskatchewan and Manitoba [2]. The sustainable production of canola is threatened, however, by the increasing prevalence of *Plasmodiophora brassicae* Woronin, a soilborne parasite that causes clubroot disease of crucifers. Clubroot development is associated with a deformation of the host root system, resulting in major yield and quality losses as water and nutrient uptake from the soil is reduced [3,4]. In western Canada, yield losses as high as 30–100% have been reported in severely infected canola crops [4].

The pathogen survives in the soil as long-lived resting spores, which under moist conditions and temperatures of 15–30 °C germinate to release primary zoospores [5,6,7]. Germination of the resting spores is enhanced by the presence of host root exudates [8,9]. The primary zoospores infect host root hairs, forming primary plasmodia from which secondary zoospores are released back into the soil. The secondary zoospores penetrate cortical root tissue and develop into intracellular secondary plasmodia, which eventually cleave to produce a new generation of resting spores [10]. It has been calculated that between 1 × 10^7^ and 1 × 10^10^ resting spores per plant can be produced over a single infection cycle [11,12,13]. Visible symptoms of clubroot appear during cortical tissue infection, when hyperplasia and hypertrophy result in formation of the root galls [10]. One of the major challenges associated with clubroot management is the persistence of *P. brassicae* resting spores in the soil. The half-life of resting spores is around four years [12,14], although some may survive for up to 17 years [14]. Recent studies from Canada have found that resting spore levels may decline by up to 90% after two years in the absence of a host crop, and that only a subset of the spores persist for much longer periods [15,16], with the pattern resembling a Type III survivorship curve [17]. 

As a soilborne pathogen, the physical and chemical conditions of the soil affect *P. brassicae* survival and infectivity, and therefore, clubroot development [18]. Alkaline soils tend to be less favorable for clubroot and are associated with milder levels of the disease [19,20,21]. This effect appears to result from reduced germination of the *P. brassicae* resting spores, declines in root hair infection, and inhibition of maturation of plasmodia, sporangia, and zoosporangia [9,22,23,24,25]. As such, liming of the soil to increase alkalinity has often been suggested as a clubroot management strategy, particularly for vegetable Brassicas grown over smaller areas [21,26]. Nonetheless, it has also been reported that acidic soils can negatively affect *P. brassicae* resting spore survival, likely to be due to stimulation of resting spore germination in absence of a host [8,27], possibly resulting in a more rapid decline in inoculum levels. 

High concentrations of nutrients such as boron, calcium, and magnesium have also been reported to reduce clubroot, but the effective quantities of those cations appear to be inversely related to soil pH [20,28,29,30,31,32]. Reductions in clubroot under high boron, calcium or magnesium concentrations have been attributed mainly to a decrease in the maturation of the primary plasmodia, which prevents the release of secondary zoospores [22,30,32]. Furthermore, the effect of calcium on clubroot has been ascribed to reduced resting spore germination and the inhibition of sporangial development and dehiscence [22,24], as well as to its involvement in the induction of defense-related compounds and *P. brassicae*-induced cell death in the host [33,34]. Boron diminishes clubroot by suppressing or delaying primary infection and cortical colonization [32,35], but its effects have been reported to be erratic and highly dependent on the soil type and the dosage, with phytotoxicity a major risk [35,36]. 

The impact of soil properties on *P. brassicae* and clubroot development are dependent on soil inoculum density, and consequently, under high inoculum concentrations, their effect is hidden [22,32]. As a result, it has been proposed that the longevity and viability of *P. brassicae* inoculum is determined by soil type, pH, ion concentration and host susceptibility, which ultimately influence pathogen inoculum density at a particular site [18]. Studies directly addressing the relationship between chemical soil properties and inoculum density of *P. brassicae* are, however, scarce. The development and implementation of improved practices for clubroot management require a better understanding of the epidemiology of this disease [37,38]. From a practical perspective, spatial epidemiological studies can help to enhance knowledge of pathogen biology and ecology, which is essential for refined and more effective sampling methods and disease management programs [37,39,40,41,42]. 

Soilborne diseases are characterized by aggregated spatial patterns, with dynamic patches that vary in size over time, and which may reflect local soil conditions that favor the disease or inhibit crop growth [39,43,44]. Observations of in-field clubroot spatial patterns have indicated that disease incidence is higher at the field entrances [45,46] or field margins [47,48], suggesting that disease spread occurs mainly through the movement of infested soil on farm machinery [45]. However, while it is widely known that clubroot tends to have a patchy spatial pattern [45,46,48], detailed evaluations of within field spread over time or the effect of soil properties on inoculum density have not been conducted. 

While maps can provide intuitive and rapid summaries of complex spatial patterns, statistical analyses are important in relating these patterns to disease dynamics [39,49,50]. Spatial autocorrelation coefficients such as Moran’s *I* are useful to measure the magnitude, intensity, and extent of spatial patterns [44,51]. Moreover, geostatistics (by the estimation of sample and fitted semi-variograms) have proven helpful in quantifying the direction, degree, and range of the spatial dependency of variables. In plant pathology, semi-variograms have been used to characterize quantitatively the spatio-temporal dynamics of plant diseases [42,52]. On the other hand, an understanding of the ecological processes and interactions that generate spatial patterns involves the estimation of statistical models able to account for spatio-temporal variation and correlation, enabling reliable inferences regarding the process under study [53,54,55]. Bayesian hierarchical modeling represents a good approach to account for variability in data sampling, spatio-temporal autocorrelation and parameter uncertainty, by partitioning complex problems into data model(s), process model(s) and parameter models [53,56]. 

The current study had two main objectives: (i) to identify and describe the spatial patterns of *P. brassicae* inoculum density and their temporal variation in four clubroot-infested fields in Alberta, Canada, where canola is regularly grown; and (ii) to evaluate the effect of soil pH, boron, calcium, and magnesium concentration on the pathogen inoculum density. Knowledge of the spatial dynamics of *P. brassicae* inoculum and the influence of soil properties on these dynamics can be useful in improving understanding of clubroot epidemiology and management.

## 2. Results

### 2.1. Inoculum Density

Between 53% and 99% of the soil samples tested negative for the presence of *P. brassicae* (Table 1). The maximum inoculum density observed differed among fields, ranging from 1.7 × 10^3^ resting spores/g soil in Field 3 (2017) to 3.2 × 10^7^ resting spores/g soil in Field 4 (2019) (Table 2). The percentage of positive samples as well as the maximum inoculum density increased from 2017 to 2019, which at first glance suggested an expansion of the infested area in all fields from one year to the other. Maximum inoculum density variation was within the same order of magnitude in Fields 1 and 2, but increased by two orders of magnitude in Fields 3 and 4 between 2017 and 2019 (Table 2).

### 2.2. Soil Chemical Properties

All fields had acidic soil, with mean pH values between 5.03 and 6.23. Mean pH in Fields 1, 2, 3, and 4 was 5.34, 5.03, 5.49, and 5.83 in 2017, respectively. In 2019, these increased to 5.96, 5.46, 5.83 and 6.23 (Table 3). Within field variation was observed in all fields. The difference between the maximum and minimum pH in Fields 1 and 4 was 2.0 in 2017 and 2.2 in 2019. In 2017, Field 3 had the same variation range as Fields 1 and 4, but in 2019 a larger difference was observed (2.9). The lowest variation was found in Field 2, where the differences were 1.5 and 1.2 in 2017 and 2019, respectively (Table 3).

Calcium, boron, and magnesium varied among fields. Mean calcium concentration in Fields 1, 2, 3, and 4 was 4648, 4129, 3853, and 4247 mg kg^−1^, respectively. Mean boron concentration was 1.97 (Field 1), 2.22 (Field 2), 1.52 (Field 3), and 2.34 mg kg^−1^ (Field 4), respectively, while mean magnesium concentration was 756.8 (Field 1), 477.6 (Field 2), 374.1 (Field 3), and 319.7 mg kg^−1^ (Field 4), respectively. Within field variation was also observed (Table 4). 

### 2.3. Spatial Patterns 

**Figure 1:**
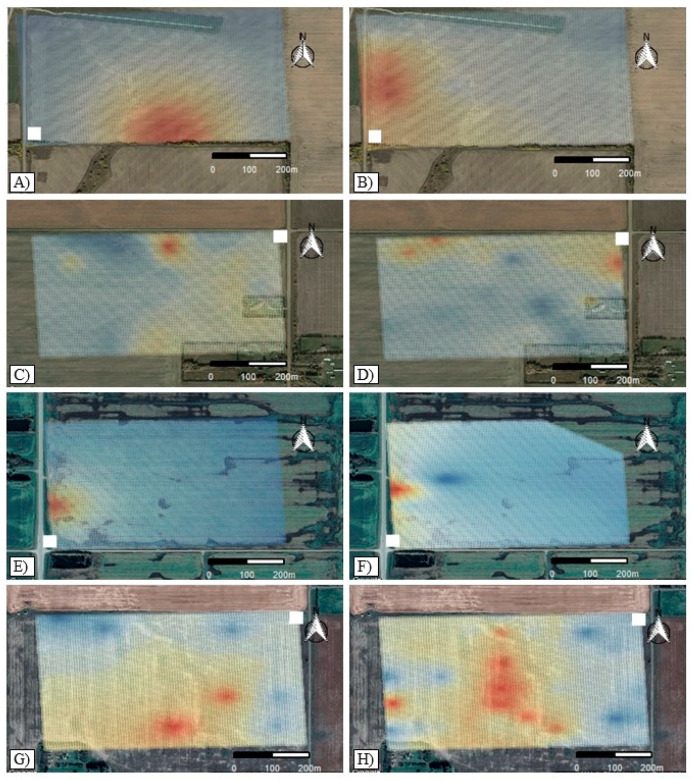
Prediction surface of the posterior mean of the log-transformed *Plasmodiophora brassicae* inoculum density for: (**A**) Field 1 in 2017; (**B**) Field 1 in 2019; (**C**) Field 2 in 2017; (**D**) Field 2 in 2019; (**E**) Field 3 in 2017; (**F**) Field 3 in 2019; (**G**) Field 4 in 2017, and (**H**) Field 4 in 2019. The white squares represent each field entrance. Color scale is relative within each field and is a representation of the *P. brassicae* inoculum density. Blue shading represents areas where *P. brassicae* was not detected, yellow shading indicates areas with intermediate inoculum densities, and red shading indicates areas with the highest inoculum densities.

Soil samples that tested positive for the presence of *P. brassicae* were located mostly at the field edges (the field edge was considered to be 10 m from the most external edge of cultivated soil) and/or adjacent to the entrance (Figure 1). Nonetheless, there was some variation in each field. For example, in Field 1 in 2017, *P. brassicae* was detected only in one sample on the southern border of the field (Figure 1A), while in 2019, most of the positive samples were on the western edge closer to the entrance (Figure 1B). In Field 2, most of the positive soil samples were identified along the northern edge, with a few positives also found along the eastern side of the field, in both 2017 and 2019 (Figure 1C,D). In Field 3, only one *P. brassicae*-infested soil sample was identified on the western edge of the field near the entrance; this patch grew in size between 2017 and 2019 (Figure 1E,F). In Field 4 in 2017, most positive samples were towards the southern edge of the field (Figure 1G), while in 2019 more positive samples were detected at the center of the field (Figure 1H).

As only one positive sample was detected in Fields 1 and 3 in 2017, it was not possible to detect spatial autocorrelation with Moran’s *I* or with the semi-variograms. The low number of positive samples also impeded the adjustment of any model to evaluate the relationship between the density of *P. brassicae* inoculum and soil properties. Therefore, for the purposes of analysis, to draw conclusions about patch growth in Fields 1 and 3, half of the distance between sampling points was regarded as the range size (40 m) for 2017. This was taken as the range size since only one sample tested positive for the presence of *P. brassicae*, and it is likely that, if samples would have been taken less than 80 m apart, the pathogen could have been detected, allowing more accurate measurement of the patch size in those fields. In Field 1, Moran’s *I* was not significant in 2019; it also was not significant in Field 4 in 2017. A small positive spatial autocorrelation was detected in Field 2 in both years and in Fields 3 and 4 in 2019. Moran’s *I* for Field 2 was 0.05 in 2017 and 0.18 in 2019, while for Fields 3 and 4 it was 0.05 and 0.06, respectively, in 2019. Positive values for this index suggest aggregation of the inoculum density to some extent, but further confirmation was required since values were close to zero.

Fitted semi-variograms indicated spatial autocorrelation in all fields with a small nugget effect (error). Structural variance *(C/(C_*0*_+C))* ranged from 77% to 100%, suggesting high spatial dependency of the inoculum density in all of the fields. In Fields 2 and 4, where semi-variograms for both years were fitted, the slope of the semi-variogram curve in the spatial range was higher in 2019 than in 2017, indicating an increase in the spatial autocorrelation. The patch diameter, measured by the spatial range, changed between years in all of the fields, with average patch growth of 221.3 m (Table 5). Patch growth in Field 3 (37.7 m) was lower than in Fields 1, 2 and 4. In Fields 1 and 2, patch growth was 249.3 m and 288.8 m, respectively. The greatest increase in patch diameter was observed in Field 4, with a patch growth of 309.3 m between 2017 and 2019.

Statistical analysis indicated a positive effect of the maximum inoculum density observed on the patch diameter (*p* = 0.015); in contrast, patch diameter was not significantly affected by the number of years when canola was grown in 2017, 2018 and 2019 (*p* = 0.308). Isotropic spread of the pathogen was observed only in Field 3. Anisotropy in Field 1 was observed at 45° (W direction). In Fields 2 and 4, it was predominant at 157.5° (NW direction), although some anisotropy was observed in Field 2 in 2019 at 90° (S direction) and in Field 4 in 2017 at 22.5° (NE direction).

Models to evaluate the effect of soil properties on inoculum density did not show an important effect of pH, boron, calcium, or magnesium on *P. brassicae* inoculum density. Means of the posterior distribution of pH, boron and calcium were erratic in all fields in both years, while for magnesium it was negative in all fields for both years, suggesting that, although this nutrient is not a critical factor defining *P. brassicae* inoculum density, it may influence it to some extent.

## 3. Discussion

Maximum *P. brassicae* inoculum densities varied from 1.7 × 10^3^ to 3.2 × 10^7^ resting spores/g soil in the fields evaluated. These values are similar to inoculum densities previously observed in commercial fields in Alberta and Europe where canola or rapeseed, respectively, was grown [15,46,47,48]. It has been reported that inoculum densities between 1 × 10^3^ and 1 × 10^5^ resting spores/g soil are sufficient to cause clubroot symptoms under field conditions [57], and that concentrations between 3 × 10^3^ and 1.3 × 10^5^ resting spores/g soil caused yield losses in susceptible hosts [48]. In the current study, *P. brassicae* resting spore numbers, as opposed to clubroot severity, were used to assess inoculum density and spatial patterns, since spore numbers are not influenced by the resistance or growing conditions of the particular crop. Nonetheless, the infestation levels observed in some of the fields suggested that significant levels of clubroot would have developed on a susceptible canola crop.

A few studies have investigated the spatial patterns of *P. brassicae* at a field level, but (to our knowledge) none has evaluated changes in these patterns over time, nor their relationship with soil chemical properties. A patchy spatial pattern has been described for the pathogen in fields in Canada, Sweden and the Czech Republic, based on anecdotal observations, descriptive statistics [45,48] and Spatial Analysis by Distance Indices (SADIE) [46,47]. Clubroot also occurs more frequently at field entrances [45,46] and margins [47]. Similarly, in the current analysis, a patchy pattern was found with respect to *P. brassicae* inoculum density in the evaluated fields, with most patches located at the field edges adjacent to the entrance. While this pattern was readily observable on the maps, based on the statistical analyses the spatial aggregation of *P. brassicae* inoculum was not as strong as expected. 

The patchiness of the *P. brassicae* inoculum was confirmed with the semi-variograms. In fields where the semi-variograms were fitted in both years, a higher spatial autocorrelation was observed in 2019 compared with 2017. Greater spatial autocorrelation was caused mainly by an increase in the number of positive samples and larger patches, which produced an increase in the extent of aggregation between samples. This phenomenon has been described in tomato crown and root rot (*Fusarium oxysporum* f. sp. *radicis-lycopersici*) [42], bell pepper crown and root rot (*Phytophthora capsici*) [40], microsclerotia of *Macrophomina phaseolina* causing root and stalk rot [58], chestnut ink disease (*Phytophthora cinnamomic* and *Phytophthora cambivora*) [59], and coffee wilt (*Fusarium xylaroides*) [52]. The limited number of soil samples that tested positive for the presence of *P. brassicae* in Fields 1 and 3 in 2017 impeded the identification of patchiness by Moran’s *I*, since autocorrelation can be detected only on a larger scale than the sampling quadrat [41,58,60]. This suggests that a lower lag distance should have been used to sample those fields during the first year to accurately detect aggregation. 

In fields where Moran’s *I* could be estimated, indices were slightly greater than zero, indicating a lack of aggregation of the inoculum density, as previously reported by Řičařová et al. (2017) [46] when estimating spatial autocorrelation using SADIE indices. This does not mean a lack of spatial autocorrelation, but rather may indicate the need for additional analysis using tools such as geostatistics, since different spatial processes can create bias in the estimation of spatial autocorrelation indices [40]. Firstly, spatial aggregation indices are sensitive to extreme observations, especially over large lag distances [58] and, secondly, those indices assume equal spatial autocorrelation in all directions (isotropy). When spatial correlation shows anisotropy, the detection of patchiness via Moran’s *I* may not be possible. Inconsistencies between Moran’s *I* and the true spatial pattern were reported by Chellemi et al. (1988) [40] in spatial analyses of *Phytophthora nicotianae* var. *parasitica* in pineapple, who suggested that the fact that inoculum was not aggregated equally in all directions could explain the discrepancies between the semi-variograms and Moran’s *I* results. 

Patch diameters ranged between 40 m and 346.1 m in 2017, consistent with a previous report by Cao et al. (2009) [45] who observed an average patch size of approximately 300 m. By 2019, the patch diameters had grown an average of 221.3 m, with diameters ranging from 77.7 m to 634.9 m. A positive relationship was identified between the maximum inoculum density and patch diameter. Collectively, the results are consistent with what was found for *M. phaseolina*, where patch growth occurred through a combination of the spread of pathogen propagules from existing infection foci and local differences in the inoculum density [58]. 

Patch growth indicates within-field pathogen dispersal. While the main mechanism for *P. brassicae* spread between fields is the movement of infested soil on farming equipment [45], within-field dispersal mechanisms are less well understood. The active dispersal of soil microorganisms through the soil matrix is generally very limited (depending on the taxon, in the order of a few millimeters to centimeters per day), although passive dispersal over larger distances is possible via accidental or specialized animal vectors, water and wind [61]. In the case of *P. brassicae*, active spread is restricted because zoospore motility is limited [62], while passive dispersal methods include movement in dust [63], via water or water-mediated soil erosion [64], and as an external contaminant of seeds and tubers [65]. The current study indicated anisotropic movement of *P. brassicae* inoculum in all fields with the exception of Field 3, where the spatial patterns were isotropic. Changes in the direction of spread can help to identify the most important pathogen dispersal methods within a field [60,66]. For example, an analysis of the direction of peak winds from April 2017 to October 2019, as recorded in weather stations surrounding the sampled fields, indicated that wind speeds >35 km/h were mostly oriented in the NW direction, corresponding to the anisotropic movement of the pathogen at 157.5° NW in Fields 2 and 4. Those results support the suggestion of Rennie et al. (2015) [63] that wind dispersal over short distances could expand resting *P. brassicae* infestations within an infested field or between immediately adjacent fields. Farming operations conducted with large equipment such as tractors and seeders could also contribute to within-field spread. The anisotropy detected at 45° in Field 1 could have reflected such operations, as was found for *Verticillium dahliae* [67] and *M. phaseolina* [68] in earlier reports.

One of the main difficulties when sampling soil pathogens is defining an appropriate sampling strategy able to capture variability between individual soil cores [69]. When defining a sampling strategy, different issues arise, among them the area to be sampled, the definition of the sampling unit, the timing of the sampling effort, and the size of sample required to answer the questions posed in relation to the pathogen populations [70]. In our research, different locations of *P. brassicae* patches were observed in Field 1 in 2017 vs. 2019. Those results may reflect large variations in and a skewed distribution of the pathogen DNA between individual soil cores [69]. Therefore, although our sampling strategy was aimed at capturing most of the within-field variation in pathogen inoculum, the sampling intensity was not enough, especially in 2017. Previous results suggest that the choice of sampling strategy is one of the main challenges associated with accurately testing a field for the presence of this pathogen [48,71]. Better and more rational sampling schemes require information on spatial patterns to evaluate both the pathogen and the disease [57,67]. Previously proposed soil sampling strategies for the detection of *P. brassicae* suggest the gathering of soil samples along a diagonal [14], or the collection of 40 subsamples in a ‘W’ transect that should be pooled in a composite sample and complemented with samples from high moisture areas, headlands and the field entrance [48]. Clubroot symptom-based sampling strategies propose evaluation for the presence of galled roots in at least 50 plants collected from a 20–30 m^2^ area near the field entrance; if symptoms are observed, further sampling is conducted along the field following a ‘W’ transect, whilst if no symptomatic plants are found, no further sampling is conducted [72]. Although previous sampling strategies may be appropriate in many cases, based on the spatial patterns observed in this study sampling should be more intense at the field edges, converging at the field entrance. 

The mean calcium, boron and magnesium levels in the sampled fields were generally consistent with what has been reported as sufficient to support adequate crop development [73,74,75]. Calcium and magnesium levels were similar to the average concentrations in soils from the Canadian prairies [73], while boron concentrations were slightly higher than average [76]. Regardless, soil pH, boron, calcium, and magnesium concentrations were not found to have an important effect on the inoculum density of *P. brassicae*. These results do not necessarily indicate that none of the soil chemical properties affect pathogen inoculum density, but rather suggest that other underlying spatial processes have a greater influence on spatial patterns. More specifically, the lack of an effect of pH on the pathogen inoculum density may reflect inconsistencies that have been reported in the relationship between pH and clubroot, since severe disease symptoms can still occur in alkaline soils under high spore loads and favorable moisture and temperature conditions [26,28,30,77]. Only weak negative correlations were found between soil pH and clubroot severity on canola in surveys of *P. brassicae*-infested fields in Alberta [26,78].

Deviations from uniform inoculum spatial patterns and disease levels may occur in homogeneous fields as a result of the temporal and spatial dynamics of the pathogen population [43]. Distinction of the spatial aggregates as a result of the population interaction versus environmental heterogeneity have been recognized in plant pathology, and those two forms of infectious disease processes have been designated as ‘truly contagious’ or ‘apparently contagious’ processes [79]. In truly contagious processes, epidemics begin from a focal point by infection of a few, often randomly spaced, individuals, and the pathogen spread results mainly from the decomposition of infected host tissue that is randomly dispersed [41]. As a result, aggregation develops around the initial infection point due to the limited dispersal of the pathogen, which generates regions of high disease prevalence around the focal point [80]. On the other hand, in apparent contagion processes, the pathogen is uniformly dispersed and randomly connected to individuals across the network, and therefore aggregation results from heterogeneity in the environment [81]. Since no effect of soil properties on the inoculum density of *P. brassicae* was observed in this study, a truly contagious process may explain the patchiness of the pathogen inoculum. 

This study indicated that *P. brassicae* inoculum occurred mostly at the field margins, converging at the field entrance. Infested patches grew each year, with this growth determined by the maximum inoculum density and active dispersal methods. In the fields evaluated, wind and possible mechanical operations contributed to in-field dispersal of the pathogen. These results suggest that adoption of field sampling strategies based on the likely spatial patterns of the pathogen, with more intense sampling towards the field edges and scattered sampling at the center of the field, may be warranted. Nonetheless, further research is required to improve understanding of the underlying processes determining *P. brassicae* spatial patterns, limit further spread of the pathogen, and optimize in-field management practices.

## 4. Materials and Methods

### 4.1. Soil Sampling

**Figure 2:**
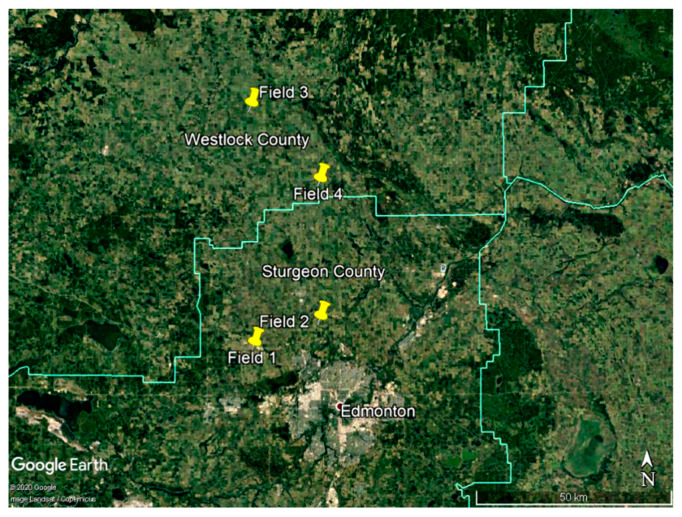
Location of fields tested for the presence of *Plasmodiophora brassicae* in 2017 and 2019 in Sturgeon and Westlock counties in central Alberta, Canada.

Four clubroot-infested fields located in Sturgeon and Westlock counties in central Alberta (Figure 2), Canada, near the center of the clubroot outbreak [82], were selected for this study. Fields 1 and 2, in Sturgeon County, had an area of 37.6 ha and 37.2 ha, respectively; Fields 3 and 4, in Westlock County, had an area of 34.6 ha and 39.2 ha, respectively. The soil in Fields 1, 2 and 4 was an Eluviated Black Chernozem, while Field 3 included three different soil types: approximately 58.7% of the field was a Gray Solodized Solonetz, 31.9% was an Eluviated Black Chernozem and the remaining 9.4% was an Orthic Humic Gleysol [83]. The crop rotation from 2017 to 2019 included canola-wheat-peas (Field 1), wheat-canola-wheat (Field 2), canola-barley-canola (Field 3) and barley-canola-oats (Field 4).

**Figure 3:**
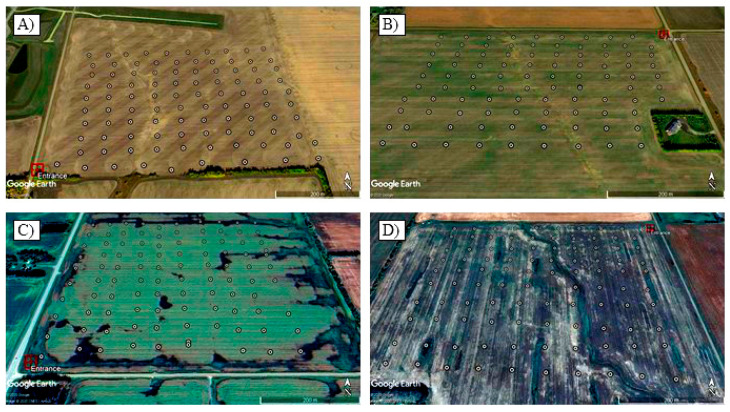
Sampling strategy in fields tested for *Plasmodiophora brassicae* inoculum in central Alberta, Canada, in 2017. The red square represents the field entrance, and each of the white points represents the location where a soil sample was collected for pathogen detection by conventional PCR and quantification by qPCR analysis in (**A**) Field 1; (**B**) Field 2; (**C**) Field 3; (**D**) Field 4.

**Figure 4:**
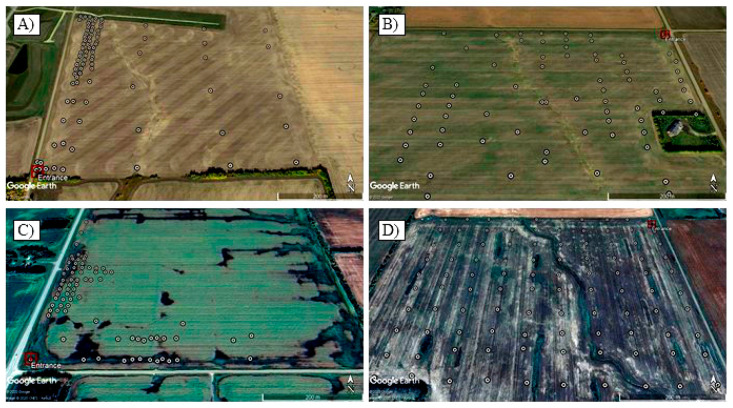
Sampling strategy in fields tested for *Plasmodiophora brassicae* inoculum in central Alberta, Canada, in 2019. The red square represents the field entrance, and each of the white points represents the location where a soil sample was collected for pathogen detection by conventional PCR and quantification by qPCR analysis in (**A**) Field 1; (**B**) Field 2; (**C**) Field 3; (**D**) Field 4.

Soil sampling in each field was conducted in October 2017 and October 2019 (Table 6). In 2017, each field was sampled extensively in a regular grid pattern (80 m × 80 m) (Figure 3), with approximately 500 g of soil collected at each node of the grids. Soils were sampled to a depth of 15 cm using a small shovel and placed individually in paper bags. All sampling locations were georeferenced with a smartphone and the geocoordinates were recorded using the mobile application MapIt Spatial [84]. Ninety-nine samples were collected from Field 1, 97 from Field 2 and 100 from each of Fields 3 and 4 (Table 6). In 2019, sampling in Fields 1, 2 and 3 was intensified around the field entrances and points that had tested positive for *P. brassicae* in 2017 (Figure 4). However, since the pathogen inoculum was more widespread in Field 4, the sampling strategy for this field was kept the same as that of 2017. Eighty-six samples were collected in Field 1, 81 in Field 2, 76 in Field 3, and 100 in Field 4 in 2019 (Table 6). 

After collection, the soil samples were air-dried and stored at 4 °C until processing. All soil samples were ground and homogenized in a mortar with a pestle or in a commercial spice grinder WSG 60 (Waring commercial, Stamford, CT, USA), which were washed with ethanol between samples. Three subsamples were taken from each homogenized soil sample, including 0.25 g for DNA extraction, 10 g for pH measurement and 200 g for nutrient quantification, as described below.

### 4.2. Soil Chemical Properties

Soil pH was measured in all samples collected in 2017 and 2019 using a commercial pH meter Orion STAR A111 (ThermoScientific, Walthman, MA, USA) with an Orion 8172BNWP Ross Sure-Flow pH electrode (ThermoScientific). Soil samples were homogenized with distilled water in solution at a 1:1 (*w*/*w*) ratio, agitated for 30 min in an oscillating table and left to settle for 30 min. Prior to taking measurements, the pH meter was calibrated with pH 4 and 7 buffer solutions. Available soil boron, calcium, and magnesium were measured in half of the samples collected in 2017, selected from each field to maintain a regular 160 m × 160 m grid. Quantification of soil nutrients was conducted by Exova Canada Inc., Edmonton, AB, Canada. Calcium and magnesium were extracted by the ammonium acetate method, while boron was extracted via the hot water method [85]. The nutrients were quantified by inductively coupled plasma optical emission spectrometry (OCP-OES). 

### 4.3. Presence and Quantity of P. brassicae in the Soil Samples

Genomic DNA was extracted from 0.25 g of each soil sample using a DNeasy PowerSoil Kit (Qiagen, Germantown, MD, USA) following the manufacturer’s instructions. The concentration and purity of the DNA were evaluated with a Nanodrop 2000c spectrophotometer (Thermo Fisher Scientific Inc., Waltham, MA, USA). Subsequently, the DNA was diluted using nuclease-free water to a concentration of 2 ng µL^−1^ for conventional PCR or diluted 10-fold for quantitative PCR (qPCR) analysis.

Conventional PCR was conducted following Cao et al. (2007) [71] with the primers TC1F and TC1R. All amplifications were carried out in a GeneAmp PCR System 9700 (Applied Biosystems, Foster City, CA, USA). Positive controls included 10 ng of *P. brassicae* DNA as a template, while 5 µL of nuclease-free water was substituted in place of the template in the negative controls. Amplicons were resolved on 2% agarose gels stained with 1X SYBR Safe (Invitrogen, Carlsbad, CA, USA). All samples that tested positive for the presence of *P. brassicae* DNA, along with all adjacent samples from the field (regardless of conventional PCR result), were evaluated further by qPCR analysis (Table 7). Quantification of the *P. brassicae* inoculum level in the soil samples was conducted by qPCR with the primers DR1F and DR1R as per Rennie et al. (2011) [65] in a StepOnePlus Real Time PCR System (Applied Biosystems, Foster City, CA, USA). Estimation of the number of resting spores per sample was completed by comparison with a standard curve generated with DNA extracted from known quantities of resting spores [65]. After each qPCR run, a melting point analysis to identify the amplified product was conducted.

### 4.4. Prevailing Wind Direction

Information regarding the prevailing wind direction from April 2017 to October 2019 was obtained from the Alberta Climate Information Service [86]. The data were collected from weather stations surrounding the sampled fields: Busby AGCM station (ID 3010979, coordinates: 53.9309, −113.9216), St. Albert Research (ID 3025750, coordinates: 53.6920, −113.6196), Legal AGCM (ID 3013790, coordinates: 54.0030, −113.4744), Dapp AGDM (ID 3061975, coordinates: 54.3290, −113.9345), and Barrhead CS (ID 3060535, coordinates: 54.1000, −114.4500).

### 4.5. Spatial Analysis

#### 4.5.1. Spatial Autocorrelation 

Statistical analyses were performed with *R* [87]. Inoculum density was log transformed prior to analysis since the distribution of this variable was highly skewed to the left [54]. Spatial autocorrelation in the *P. brassicae* inoculum density was evaluated by Moran’s *I*, estimated using the package *spdep* [88]. Moran’s *I* is a classic correlation index that ranges from −1 to 1, the absolute value of which increases with the autocorrelation; values near 0 indicate an absence of spatial autocorrelation, positive values indicate positive autocorrelation, and negative values indicate negative autocorrelation [89].

Experimental semi-variograms for each field in both sampling years were generated by plotting semi-variance versus lag distance (distance between pairs at which the semi-variogram is calculated) with the package *gstat* [90]. The presence or absence of anisotropic patterns was determined by examination of the semi-variograms at 0°, 22.5°, 45°, 67.5°, 90°, 112.5°, 135° and 157.5°, where 0° represents the N direction. Semi-variograms represent the average squared differences in values between pairs of samples, and include statistical information on the observed differences between values, depending on the distance between individuals [89]. Semi-variance values that increase with lag distance indicate spatially dependent samples [66]. Afterwards, a spherical model was fitted to each sample semi-variogram using the least square approach of Cressie (1993) [91]. Fitted semi-variograms allow for the description of spatial patterns through the estimation of the autocorrelation parameters: (i) the spatial range, which indicates the maximum distance at which spatial autocorrelation is present and can be regarded as an index of the average patch diameter [92]; (ii) the nugget (*C*_0_), which is the estimate of the error in the measurements and environmental variability; and (iii) the sill (*C_*0*_+C*), which quantifies the spatial pattern intensity [51]. 

#### 4.5.2. Models 

A Bayesian hierarchical spatial approach was used to model the relationship between *P. brassicae* inoculum density and soil pH, and concentration of boron, calcium, and magnesium in the soil. In this approach, a stochastic spatial effect was added to a generalized linear model. Hurdle models (also known as Zero-Altered Poisson models) were fitted since the data presented a problem of zero-inflation, and we aimed to identify the possible effect of each covariate on the presence/absence of the pathogen and, when it was present, on the number of *P. brassicae* resting spores [93]. Misalignment of soil nutrient concentrations and resting spore numbers occurred, since boron, calcium, and magnesium were quantified in half of the points where *P. brassicae* inoculum was quantified; therefore, spatial variation in the nutrient concentration and the number of resting spores were modelled jointly [94]. Posterior distributions for model parameters were approximated with the Integrated Nested Laplace Approximation using the package *INLA* [95]. The fitted model can be written as:

(1) $$RS_{i}∼ZAP\left(\mu_{i},\pi_{i}\right)$$

(2) $$E\left(RS_{i}\right)=\pi \left(\frac{\mu_{i}}{1-e^{-\mu^{i}}}\right)$$

(3) $$var\left(RS_{i}\right)=\frac{\pi_{i}}{1-e^{-\mu^{i}}}\left(\mu_{i}+\mu_{i}^{2}\right)-{\left(\frac{\mu_{i}\pi_{i}}{1-e^{-\mu^{i}}}\right)}^{2}$$

(4) $$log\left(\mu_{i}\right)=\beta_{1}+\beta_{2}pH_{i}+\beta_{3}Ca_{i}+\beta_{4}B_{i}+\beta_{5}Mg_{i}+u_{i}$$

(5) $$logit\left(\pi_{i}\right)=\gamma_{1}+\gamma_{2}pH_{i}+\gamma_{3}Ca_{i}+\gamma_{4}B_{i}+\gamma_{5}Mg_{i}+v_{i}$$

where, $RS_{i}$ is the logarithm of the number of *P. brassicae* resting spores per g of soil; $ZAP\left(\mu_{i},\pi_{i}\right)$ corresponds to a Zero-Altered Poisson distribution with parameters $\mu_{i}$ and $\pi_{i}$; $\mu_{i}$ is the population mean and $\pi_{i}$ is the probability of the presence of *P. brassicae*; $\beta_{1}$ is the intercept for the count (Poisson) component of the model; $\gamma_{1}$ is the intercept for the binary component of the model (presence/absence of *P. brassicae*); $\beta_{2,3,4,5}$ are the estimators for each covariate in the count part of the model; $\gamma_{2,3,4,5}$ are the estimators for each covariate in the binary part of the model; pH is the soil pH; B, Ca and Mg are the concentrations (mg kg^−1^) of boron, calcium and magnesium in the soil; and $u_{i}$ and $v_{i}$ are spatially correlated random effects in the count and binary parts of the model, respectively. The spatial terms $u_{i}$ and $v_{i}$ were assumed to have a multivariate Gaussian distribution whose covariance matrix depended on the distance between locations. The spatial fields were resolved with the explicit link between Gaussian Markov random fields and continuous Gaussian fields with a Matérn covariance structure via a weak solution to a stochastic partial differential equation (SPDE) [96]. 

Models including all possible combinations among covariates were compared using the Watanable Akaike Information Criterion (WAIC) and the Deviance Information Criterion (DIC) [93]. Hypothesis testing was conducted by checking whether zero fell within the 95% Bayesian credibility interval (CI) of the parameter estimators [97]. If zero fell in the CI, the null hypothesis was accepted, and therefore the covariate was not assumed to have an important effect on *P. brassicae* inoculum density. Prediction surfaces of the mean of the posterior distribution of *P. brassicae* inoculum density were mapped using the packages *ggplot2* [98] and *ggmap* [99]. Additional modeling was conducted using the *nlme* package [100] to test, by a frequentist approach, the effect of maximum inoculum density and rotation scheme on clubroot patch diameter. Results from both the frequentist and Bayesian approach were compared.

## Figures and Tables

**Table 1:** Number and percentage of soil samples that tested negative for the presence of *Plasmodiophora brassicae* DNA by conventional PCR. Soil samples were collected from four clubroot-infested fields located in Sturgeon and Westlock counties in central Alberta, Canada.

Field/Year	2017			2019		
	Number of Samples Collected	Number of Negative Samples	Percentage of Negative Samples	Number of Samples Collected	Number of Negative Samples	Percentage of Negative Samples
Field 1	99	98	99%	86	73	85%
Field 2	97	74	76%	81	43	53%
Field 3	100	99	99%	76	68	89%
Field 4	100	72	72%	100	53	53%

**Table 2:** Minimum and maximum *Plasmodiophora brassicae* inoculum density quantified by qPCR in soil samples collected from four clubroot-infested fields located in Sturgeon and Westlock counties in central Alberta, Canada.

Field/Year	2017		2019	
	Minimum (Resting Spores/g Soil)	Maximum (Resting Spores/g Soil)	Minimum (Resting Spores/g Soil)	Maximum (Resting Spores/g Soil)
Field 1	na *	1.4 × 10^5^	4.3 × 10^3^	2.7 × 10^5^
Field 2	1.1 × 10^4^	1.7 × 10^7^	6.7 × 10^3^	9.9 × 10^6^
Field 3	na	1.7 × 10^3^	4.3 × 10^3^	1.7 × 10^5^
Field 4	5.4 × 10^3^	1 × 10^5^	5 × 10^3^	3.2 × 10^7^

* na, not applicable.

**Table 3:** Minimum, maximum, and mean pH of soil samples collected from four clubroot-infested fields located in Sturgeon and Westlock counties in central Alberta, Canada. Soil pH was measured in a soil water: solution ratio 1:1 (*w*/*w*).

pH	Year	Min	Max	Mean
Field 1	2017	4.7	6.72	5.34
	2019	4.94	7.14	5.96
Field 2	2017	4.49	5.97	5.03
	2019	4.83	6.05	5.46
Field 3	2017	4.64	6.67	5.49
	2019	4.45	7.32	5.83
Field 4	2017	4.93	6.95	5.83
	2019	5.24	7.42	6.23

**Table 4:** Minimum, maximum, and mean concentration of calcium, boron, and magnesium in soil samples collected in 2017 from four clubroot-infested fields located in Sturgeon and Westlock counties in central Alberta, Canada.

Field	Element	Min (mg kg^−1^)	Max (mg kg^−1^)	Mean (mg kg^−1^)
Field 1	Ca	2990	6470	4648
Field 2		3440	5880	4129
Field 3		2090	5140	3853
Field 4		3780	6190	4247
Field 1	B	0.97	3	1.969
Field 2		1.6	3	2.222
Field 3		0.79	2.7	1.515
Field 4		1.2	4.2	2.336
Field 1	Mg	432	1200	756.8
Field 2		291	681	477.6
Field 3		214	513	374.1
Field 4		159	614	319.7

**Table 5:** Semi-variogram parameters describing the spread of the log-transformed *Plasmodiophora brassicae* inoculum density in four clubroot-infested fields located in Sturgeon and Westlock counties in central Alberta, Canada.

Field	Year	Nugget (*C_*0*_*)	Sill (*C+C_*0*_*)	*C/(C+C_*0*_)*	Range (m)
Field 1	2017 *	NA	NA	NA	40.0 ^†^
	2019	0.01	0.20	0.96	289.2
Field 2	2017	0.12	0.72	0.83	346.1
	2019	0.00	4.00	1.00	634.9
Field 3	2017 *	NA	NA	NA	40.0 ^†^
	2019	0.00	0.10	1.00	77.7
Field 4	2017	0.00	0.912	1.00	113.6
	2019	0.58	2.49	0.77	422.9

* Identification of only one positive sample did not allow fitting of a semi-variogram, and hence parameters are not presented; NA, not applicable. ^†^ When only one positive sample was observed, half of the distance between sampling points was assumed as the range (40 m).

**Table 6:** Sampling dates, locations and number of samples collected in each of four fields sampled in central Alberta, Canada, in 2017 and 2019.

Field	County	Date of Sampling	Number of Collected Samples
Field 1	Sturgeon County	12 October 2017	99
		17 October 2019	86
Field 2	Sturgeon County	12 October 2017	97
		17 October 2019	81
Field 3	Westlock County	13 October 2017	100
		18 October 2019	76
Field 4	Westlock County	13 October 2017	100
		18 October 2019	100

**Table 7:** Number of samples that tested positive for the presence of *Plasmodiophora brassicae* DNA by conventional PCR and number of samples where the pathogen inoculum density was quantified by qPCR.

Field	Year	Number of Positive Samples	Number of Samples Quantified for Inoculum Density
Field 1	2017	1	6
	2019	13	40
Field 2	2017	23	45
	2019	38	54
Field 3	2017	1	5
	2019	8	33
Field 4	2017	28	53
	2019	47	65

## Data Availability

All raw data are available and provided upon request.
